# Dental caries status and its associated factors among 5-year-old Hong Kong children: a cross-sectional study

**DOI:** 10.1186/s12903-017-0413-2

**Published:** 2017-08-31

**Authors:** Kitty Jieyi Chen, Sherry Shiqian Gao, Duangporn Duangthip, Samantha Kar Yan Li, Edward Chin Man Lo, Chun Hung Chu

**Affiliations:** Faculty of Dentistry, The University of Hong Kong, 34 Hospital Road, Hong Kong, Hong Kong, Special Administrative Region of China

**Keywords:** Caries, Oral health, Children

## Abstract

**Background:**

This study investigated dental caries status and its associated factors among 5-year-old children in Hong Kong.

**Method:**

This cross-sectional survey was conducted in 2016. It comprised a questionnaire survey and a clinical examination. Kindergarten children aged 5 were recruited using a multistage sampling method. Parents of the participating children were asked about their children’s demographic information, sugary snacking behaviours, and oral health–related behaviours and about their own oral health knowledge. One trained dentist performed oral examinations on the children. Caries experience was measured using the dmft index. The relationships between the dmft scores and background information, sugary snacking behaviours, oral health–related behaviours and parental dental knowledge were studied using a zero-inflated negative binomial (ZINB) regression analysis.

**Results:**

A total of 570 children were invited to participate, and 501 completed the oral examination (response rate: 88%). The prevalence of dental caries was 55%, and the mean dmft score was 2.7 ± 3.7. Decayed teeth (dt) constituted 93% of caries experience. ZINB analysis found that children who visited a dentist, who were taken care of primarily by grandparents and whose parental dental knowledge levels were moderate had higher dmft scores. Children who ate sugary snacks more than twice daily, had irregular dental attendance and lived in low-income families had a significantly higher chance of having dental caries.

**Conclusions:**

Dental caries was prevalent among 5-year-old Hong Kong children, and most of the decayed teeth were untreated. The caries prevalence of the children was related to their frequency of sugary snack intake, dental attendance and socio-economic background.

**Electronic supplementary material:**

The online version of this article (10.1186/s12903-017-0413-2) contains supplementary material, which is available to authorized users.

## Background

Dental caries is a common oral disease affecting preschool children around the world [1]. The development of this multifactorial disease is affected not only by cariogenic plaque, fermentable carbohydrates, susceptible host (tooth) and time, but also by environmental factors such as saliva and availability of fluoride [2]. A literature review reported that the prevalence of dental caries has increased in both developed and developing countries in the past decade [3]. Dental caries causes pain as well as local and systematic infection. It will progress into tooth pulp and a dental abscess will form if untreated. Moreover, it affects children’s nutrition, growth and development, general health and quality of life [4].

The water supply in Hong Kong has been fluoridated since 1961, and is currently maintained at 0.5 ppm fluoride [5]. Apart from water fluoridation, the Department of Health has set up an Oral Health Education Unit and implemented preventive programs to reduce dental caries among preschool children. An oral health education program for kindergarten children has been carried out since 1993. The program promotes good oral health–related behaviours among kindergarten children. The Oral Health Education Unit also has organized educational programs to increase kindergarten teachers’ and parents’ oral health knowledge. Educational materials including posters, pamphlets and a website called ‘Tooth Club’ have been developed to promote oral health to various sectors of the community through specifically targeted oral health promotion programmes. The Tooth Club also disseminates oral health messages and promotes awareness of oral health, oral hygiene practices and the proper use of oral health care services in Hong Kong.

In general, the experience and prevalence of caries among preschool children in Hong Kong decreased after the introduction of water fluoridation [6]. An epidemiological survey conducted by the World Health Organisation (WHO) in 1968 found that the caries incidence based on mean dmft score of 5- to 6-year-old children was 5.3 [7]. In a second survey conducted by the Faculty of Dentistry, The University of Hong Kong in 1991 [8], the results showed that the mean dmft score of 5-year-old Chinese children was 3.2. Two more surveys conducted in 1999 and 2009 by the university [6, 9] found that the mean dmft score of 5-year-old children was 1.8 in 1999 and 2.2 in 2009. These two surveys also reported that about half of the 5-year-old preschool children had caries experience, and the great majority of these cases of decay (>90%) were untreated. The results of these surveys suggested that the caries status of Hong Kong preschool children experienced no significant change over the years.

According to the recommendation of the WHO, epidemiological surveys should be carried out at five-year intervals to monitor the oral health status in a community [10]. This is particularly essential in Hong Kong due to the high caries prevalence of preschool children. Because the last territory-wide survey on the oral health status of 5-year-old children was conducted in 2009 [9], another survey should have been performed by 2014. The results of the survey would be useful for dental professionals and governments in planning dental services for preschool children. This study aimed at investigating the experience and prevalence of caries among 5-year-old children in Hong Kong. In addition, the study reported the common factors associated with dental caries.

## Method

This study was conducted in 2016 with ethical approval from the Institutional Review Board of the University of Hong Kong/ Hospital Authority Hong Kong West Cluster (IRB: UW 16–180).

Written consent was obtained from the parent of each child.

### Sample size calculation and sample selection

Caries prevalence was estimated to be 49% [9]. The confidence level was set at 95%, and the confidence interval was set at 5% (CI: 44 to 54%). The sample size required was 384. With an estimated response rate of 80%, at least 480 children needed to be invited to participate in this study.

A multistage sampling method was adopted in this study. Hong Kong’s three main geographic regions, namely Hong Kong Island, Kowloon and the New Territories, were regarded as clusters. The ratio of children invited from the three clusters was determined according to the ratio of distribution of the population [11]. Registered kindergartens in each cluster (district) were numbered sequentially. They were chosen by a simple random sampling method using a list of random number generated by a computer. All 5-year-old children in the selected kindergartens were invited to the survey. Kindergartens were invited until the invited number of subjects in the cluster was fulfilled. The inclusion criterion of this study was generally healthy children. Children who had special needs, had undergone prolonged use of medications or had severe chronic diseases were excluded from this study.

### Questionnaire survey

A consent form and questionnaire were sent to parents of the children. The questionnaire was adapted from one used in the previous surveys [6, 9] (Additional file 1), and it consisted of four parts as follows:

1. the child’s background information: sex, age, birthplace, parenthood and his/her primary caregiver;
2. the child’s socio-economic information: family income and parents’ education levels;
3. the child’s oral health–related behaviours: bottle-feeding habits, sugary snacking habits, toothbrushing habits and dental attendance experience; and
4. the child’s parent’s dental knowledge: Twenty-one multiple-choice questions adapted from previous surveys for parents of Hong Kong preschool children asked about the cause and prevention of dental diseases. For each correct answer, a score of 1 was given, whereas no score was given for a wrong or ‘don’t know’ answer. Therefore, the total score ranging from 0 to 21 was classified into one of three levels: low (score 0–7), moderate (score 8–14) or high (score 15–21).

The returned questionnaires were checked by an assistant who followed up missing and inappropriate nswers on the questionnaire by phone.

### Clinical examination

One trained dentist performed the clinical examination in a kindergarten classroom using a disposable dental mirror with a light-emitting diode (LED) light and ball-end Community Periodontal Index (CPI) probe. Food debris was gently removed to avoid under recording of dental caries. The diagnostic criteria for caries followed the recommendation of the WHO [10]. Caries experience was measured with the dmft index. A tooth was recorded as decayed (dt) when a lesion had an unmistakable cavity, undermined enamel, or detectably softened floor or wall. A tooth was recorded as missing (mt) when it was extracted due to caries. A tooth was recorded as filled (ft) when it was permanently filled without caries The examiner (KJC) was calibrated with an experienced dental epidemiologist (DD) in the same setting before the study. The intra-examiner agreement was assessed through re-examining a 10% random sample of the children on the same day. The selection of children for duplicate examination was performed by a dental assistant without notifying the examiner. After the dental examination, an individual child’s oral health report was submitted to the parent of each child. Following the proposal approved by the Institutional Review Board, no dental treatment was provided on site. Parents could seek further dental treatment by their own cost if necessary.

### Statistical analysis

Data analysis was performed using Statistical Package for Social Science version 22.0 (SPSS Inc., Chicago, Illinois, USA), STATA version 14 (StataCorp, College Station, Texas, USA) and SAS OnDemand for Academics (SAS Institute Ltd., North Caroline, USA). Children with missing data were excluded from the analysis. Statistical sample weights were performed. The intra-examiner agreement was assessed using Kappa statistics. A chi-square test was used to test the association of caries experience with the variables studied. The Mann-Whitney U test or Kruskal-Wallis H test was employed to study the distribution of dmft scores according to different variables. Independent variables with *p*-values of less than 0.10 for the chi-square test, Mann-Whitney U test and Kruskal-Wallis H test were studied as covariates in the regression model. The Poisson model, the negative binomial model and zero-inflated models were taken into account to study the relationships between the dmft scores and selected variables. Vuong’s test was employed to choose an appropriate model. Backward stepwise selection was used to remove insignificant variables from the regression model. The selection was completed when all of the remaining variables had a *p*-value less than 0.05. The level of significance for all tests was set at 0.05.

## Results

A total of 570 children from seven kindergartens were invited to participate, and 501 children completed the oral examination. The response rate was 88% (501/570). The percentages of non-respondents in Hong Kong, Kowloon and New Territories were 3% (*n* = 3), 3% (*n* = 6) and 21% (*n* = 60), respectively. The main reason for non-participation was lack of parental consent (60 children, 60/69, 87%), and nine children (9/69, 13%) were absent from school on the day of the examination. Among these 501 children, there were 42 students without a complete questionnaire. Seven children were from Hong Kong (7/88, 8%), 15 were from Kowloon (15/187, 8%) and 20 were from New Territories (20/226, 9%). There was no significant difference in caries prevalence (dmft > 0) and median dmft score between children with and without a complete questionnaire (53% vs. 45%; 2.5 ± 3.6 vs. 2.7 ± 3.9). The result showed a nonrepresentation of children from New Territories. The ratio of recruited children in Hong Kong Island, Kowloon and the New Territories was 2:4:5, whereas the population ratio is approximately 2:4:8. Therefore, proportional sample weights were performed on the 459 children with a completed questionnaire. The following descriptive data and results of analysis were weighted.

**Fig. 1:**
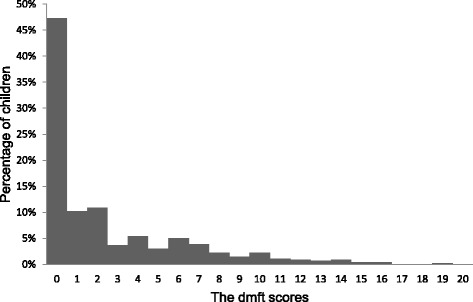
Caries experience (dmft) of the 5-year-old children (*n* = 459)

Among the children in the study, 210 (46%) were boys, and 425 (93%) were born in Hong Kong. There were 254 (55%) children with caries experience (dmft > 0). The mean dmft score (±SD) was 2.7 ± 3.7. Untreated decayed teeth (dt = 2.6 ± 3.7) represented 93% of the dmft score (Table 1). The Kappa value for the assessment of caries status was 0.97. The distribution of the dmft score was positively skewed, with a skewness of 1.7 (Fig. 1). The prevalence of caries experience in maxillary teeth was higher than that in mandibular teeth. Maxillary incisors had the highest prevalence of caries (31.6%), whereas mandibular incisors had the lowest prevalence of caries (0.7%). However, mandibular molars had a higher caries prevalence rate than maxillary molars.

**Table 1:** Caries experience according to sex and birthplace

Independent variables (n)	Caries prevalence	p-value^1^	Mean dmft (±SD)	Mean dt (±SD)	Mean mt (±SD)	Mean ft. (±SD)	Rank of median dmft score	p-value^2^
All children (459)	55.4%		2.7 ± 3.7	2.6 ± 3.7	0.01 ± 0.1	0.2 ± 0.8		
Sex		0.157						0.056
Boys (210)	59.1%		2.9 ± 3.9	2.9 ± 4.0	0.01 ± 0.1	0.2 ± 0.8	243	
Girls (249)	52.2%		2.2 ± 3.3	2.2 ± 3.3	<0.01	0.1 ± 0.7	220	
Birthplace		0.477						0.219
Hong Kong (425)	54.8%		2.5 ± 3.6	2.5 ± 3.7	0.01 ± 0.1	0.2 ± 0.8	228	
Outside of Hong Kong (34)	61.8%		3.0 ± 3.6	2.7 + 3.6	<0.01	0.2 ± 1.1	255	

^1^Comparison of caries prevalence

^2^Comparison of the rank of median dmft score

Most of the parents (84%) reported that their children had stopped using a night-time bottle. A small number of the children (16%) still fed with a bottle before sleeping. Many of the children (43%) had sugary snacks twice or more daily. More than half of the children (66%) brushed their teeth more than twice daily. Parents reported that a considerable proportion of the children (31%) started brushing after 24 months, and most of them brushed with parental assistance (70%). A small number of the children (16%) adopted other cleansing aids, such as dental floss. It was also found that a majority of the children (75%) had no dental attendance experience (Table 2).

**Table 2:** Dental caries prevalence (dmft > 0) and independent variables

Variables (*n* = 459)	Dental caries prevalence	*p*-value	Pairwise comparison
Bottle feeding before sleeping		0.900	
Yes (75)	56.0%		
No (384)	55.1%		
Bottle feeding duration		0.853	
≤ 24 months (184)	56.0%		
> 24 months (202)	54.0%		
Still fed with bottle (73)	57.5%		
Frequency of sugary snack intake (times per day)		0.002	
≤ 2 (260)	46.6%		
> 2 (199)	61.0%		
Brushing frequency (times per day)		0.197	
< 2 (154)	59.7%		
≥ 2 (305)	53.3%		
Brushing started age (months)		0.474	
0–24 (319)	54.2%		
After 24 (140)	58.3%		
Use other cleansing aids		0.305	
Yes (72)	61.1%		
No (387)	54.4%		
Assistance in brushing		0.602	
Yes (328)	56.3%		
No (131)	53.1%		
Dental attendance		<0.001	a < b
Yes, regularly (33) ^a^	50.0%		
Yes, irregularly (80) ^b^	77.2%		
No (346) ^a^	51.2%		
Monthly family income (HKD)		0.001	b < a
≤ 15,000 ^a^ (177)	65.0%		
15,001–30,000 ^a, b^ (168)	54.2%		
≥ 30,001 ^b^ (114)	42.0%		
Father’s education level		0.547	
Primary or below (32)	58.1%		
Secondary (274)	57.3%		
Tertiary or above (153)	52.0%		
Mother’s education level		0.027	b < a
Primary or below ^a^ (44)	72.7%		
Secondary ^a, b^ (256)	55.7%		
Tertiary or above ^b^ (159)	50.0%		
Parental dental knowledge level		0.006	a < b
Low ^a, b^ (25)	72.0%		
Moderate ^b^ (259)	59.8%		
High ^a^ (175)	46.6%		
Parenthood		1.000	
Both parents (406)	55.8%		
Single parent or other (53)	55.3%		
Primary caregiver		0.025	a < b
Parents ^a, b^ (322)	55.0%		
Grandparents or relatives ^b^ (97)	63.9%		
Domestic helpers ^a^ (40)	38.5%		

In the bivariate analysis, there was no statistically significant difference in caries prevalence between boys and girls (*p* = 0.157), or between children born in Hong Kong and outside of Hong Kong (*p* = 0.477) (Table 1). Children who ate sugary snacks twice or more daily had higher caries prevalence (*p* = 0.002) than those who did not. Children with previous irregular dental attendance had a higher prevalence of caries than those who had never or regularly visited a dentist (*p* < 0.001). Higher caries prevalence was found in children who did not have high parental dental knowledge levels than in those with high parental knowledge levels (*p* = 0.006). Moreover, children whose mothers had received primary education or below had higher caries prevalence than those whose mothers had higher education levels (*p* = 0.027). Furthermore, a high proportion of children with caries experience was found in families with incomes of less than HK$ 15,000 per month (*p* = 0.001) and in which the children were cared for by grandparents (*p* = 0.025) (Table 2). In the Mann-Whitney U test and Kruskal-Wallis H test, a higher rank of median dmft score was found for children who had previous irregular dental attendance, had a low level of parental dental knowledge, had low family income, had low maternal education levels, ate sugary snacks twice or more daily and were primarily cared for by grandparents (Table 3).

**Table 3:** Median dmft scores and independent variables

Variables (*n* = 459)	Rank of median dmft score	*p*-value	Pairwise comparison
Bottle feeding before sleeping		0.420	
Yes	241		
No	228		
Bottle feeding duration		0.592	
≤ 24 months	226		
> 24 months	229		
Still fed with bottle	244		
Frequency of sugary snack intake (times per day)		0.001	
≤ 2	213		
> 2	253		
Brushing frequency (times per day)		0.124	
< 2	243		
≥ 2	224		
Brushing started age (months)		0.144	
0–24	225		
After 24	243		
Use other cleansing aids		0.505	
Yes	239		
No	228		
Assistance in brushing		0.510	
Yes	233		
No	224		
Dental attendance		<0.001	a < b
Yes, regularly ^a^	225		
Yes, irregularly ^b^	301		
No ^a^	214		
Monthly family income (HKD)		<0.001	b < a
≤ 15,000 ^a^	258		
15,001–30,000 ^a, b^	230		
≥ 30,001 ^b^	195		
Father’s education level		0.536	
Primary or below	236		
Secondary	235		
Tertiary or above	221		
Mother’s education level		0.025	b < a
Primary or below ^a^	272		
Secondary ^a, b^	234		
Tertiary or above ^b^	214		
Parental dental knowledge level		0.003	b < a
Low ^a, b^	277		
Moderate ^b^	242		
High ^a^	208		
Parenthood		0.582	
Both parents	229		
Single parent or other	239		
Primary caregiver		0.009	a < b
Parents ^a, b^	228		
Grandparents or relatives ^b^	257		
Domestic helpers ^a^	189		

The result of Vuong’s test showed that the zero-inflated negative binomial (ZINB) model fit the data better (*p* < 0.001). The ZINB regression model showed that dental attendance was positively associated with the dmft score in the negative binomial part (incidence risk ratio [IRR] = 1.77 for regular dental attendance, and 1.91 for irregular dental attendance). In addition, the results from the zero-inflated part indicated that children who had irregular dental attendance had a lower chance of having ‘no caries experience’, i.e. being ‘an excess zero’ (odds ratio [OR] = 0.28), than did those who had no dental attendance experience. The zero-inflated part implied that children who ate sugary snacks less than twice daily had a higher probability of having ‘no caries experience’ (OR = 1.98). Children from high-income families had a higher chance of having ‘no caries experience’ compared to those from low-income families (OR = 3.35). In the negative binomial model, the dmft score was associated with having a moderate parental dental knowledge level (IRR = 1.51) and being taken care of by grandparents (IRR = 1.33) (Table 4).

**Table 4:** Caries risk factors of the 5-year-old children (zero-inflated model, *n* = 459)

Zero-inflated portion (dmft = 0)	Variables	OR	*p*-value	95% C.I.
	Frequency of sugary snack intake		0.010	
	≤2 per day	1.982		1.177–3.336
	>2 per day*			
	Dental attendance experience		0.005	
	Yes, regularly	0.749	0.512	0.315–1.779
	Yes, not regularly	0.276	0.001	0.127–0.595
	No *			
	Family monthly income		0.002	
	≥ HK$ 30,001	3.353	<0.001	1.725–6.521
	HK$ 15,001–30,000	1.675	0.100	0.907–3.096
	HK$ ≤15,000*			
Negative binomial portion (dmft > 0)	Variables	IRR	*p*-value	95% C.I.
	Dental attendance experience		<0.001	
	Yes, regularly	1.772	0.001	1.267–2.480
	Yes, not regularly	1.909	<0.001	1.495–2.438
	No *			
	Parental dental knowledge		0.005	
	Low	1.195	0.283	0.863–1.653
	Moderate	1.508	0.001	1.171–1.942
	High*			
	Primary caregiver		0.026	
	Grandparents	1.334	0.034	1.022–1.742
	Domestic helpers	0.690	0.067	0.466–1.027
	Parents*			

*Reference group

## Discussion

This study was reported following the Statement of Strengthening the Reporting of Observational Studies in Epidemiology (STROBE) [12]. This statement provides a checklist of items for a systematic report. However, the checklist is not an instrument to evaluate the quality of this study. The results of this study showed that the experience and prevalence of caries among 5-year-old children in Hong Kong has not significantly changed since 1999 [6]. It also showed that the oral health status of Hong Kong preschool children does not achieve the goal set by the WHO and the World Dental Federation in 2000 that more than half of children should have no caries experience [13].

The caries status of Hong Kong preschoolers is worse than that of Singaporean children. Singapore is considered similar to Hong Kong in terms of economic development and water fluoridation [14]. The government in Singapore places a strong emphasis on caries prevention and on inculcating good oral hygiene practices for children at an early age. Subsidized dental care for preschool children is available at the Health Promotion Board’s School Dental Centre. The prevalence of dental caries was also high when compared to the United Kingdom, which provides free comprehensive dental care to preschool children [15]. Prevention and simple treatment should be undertaken at an early age, when intervention is most cost-effective [5]. The Department of Heath should consider implementing dental care service to improve dental health for preschool children in Hong Kong.

Kindergarten education is highly subsidised, and the great majority of children attend preschools in Hong Kong. Homeschooling is uncommon. This study employed a multistage sampling method to recruit 5-year-old children enrolled in kindergarten. This sampling method was efficient for obtaining samples to represent the general child population to some extent, saving both time and resources. However, it is unknown whether the child population was directly proportional to the total population of the three geographic regions, and hence this sampling method could be considered arbitrary. Moreover, because this multistage sampling method, it out portions of the child population from the study, the study’s findings could be different from those of a study using random sampling and may not be completely representative of the child population.

Weighting adjustment of the sample was performed because the ratio of recruited children in the three clusters was different from the ratio of the population. The ratio of recruited males to females was consistent with the ratio of the general population [11]. The response rate was satisfactory. One of the main reasons for this was good cooperation with the kindergarten teachers, who helped in promoting this survey to the parents.

The dmft index is a commonly used index for epidemiological studies in dental research [16]. It is a simple count of decayed, missing and filled teeth. The count data, namely non-negative values, include zero and positive integers [17]. Previous oral health surveys showed that the distribution of dental caries among 5-year-old children in Hong Kong was overdispersed with excess zero (dmft = 0). The normality assumption could not be held when analysing the data. Non-parametric methods, including the Mann-Whitney U test or Kruskal-Wallis H test, were employed to study the distribution of dmft scores according to different variables [18].

Vuong’s test was employed in this study to choose a suitable model [19]. The results showed that the ZINB regression model fit the data of this study better than other common statistical models. Thus, ZINB regression analysis was used to study the effect of independent variables on the dmft score. The zero-inflated part of the model showed the relationship between covariates and the presence of caries. The negative binomial part of the model indicated the association between covariates and positive caries experience (dmft > 0) [16]. Variables with *p*-values of less than 0.10 in univariate analysis were considered covariates in the regression model. Doing so can minimise the influence of irrelevant variables and prevent missing important variables.

The present study found that sex was not associated with dental caries. These findings were consistent with previous studies [6, 9]. A study found that night-time use of a bottle may be associated with early childhood caries [20]. However, this study and the two previous studies in Hong Kong could not find an association between night-time use of a bottle and caries prevalence. Although surveys reported in 1999 and 2012 showed that the birthplace of preschool children was associated with caries experience, this study found that the experience and prevalence of caries was similar among children whether they were born in or outside Hong Kong.

The present study found an association between dental attendance and caries experience as well as caries prevalence. A reason for the poor dental health of preschoolers with irregular dental attendance might be their problem-orientated dental care–seeking behaviour [21]. Therefore, an oral health promotion campaign is necessary to improve the poor attitude and awareness towards dental health of preschoolers and their parents. This study found that high monthly family income reduced the risk of having dental caries. Moderate parental dental knowledge levels as well as having grandparents as the primary caregivers were factors that increased caries experience [22]. Parents with high incomes may be well educated and have high dental knowledge levels, which might explain the better oral health of their children. A study reported that children who are primarily taken care of by grandparents can be indulged with sugary foods [23].

Children who ate sugary snacks twice or more daily had higher caries experience than those who did not. Dietary analysis and advice are important when clinicians are formulating treatment plans for their child patients. Refined sugar is readily available in Hong Kong, and the excessive intake of sugar in traditional foods, desserts and soft drinks has created much discussion in the community. Legislation has been implemented that all foods, including snacks and beverages, must have nutritional labels to indicate their sugar content. Policies regarding a sugar tax and warning labels about sugar intake have also been proposed.

## Conclusion

In conclusion, the experience and prevalence of caries remained high among 5-year-old children in Hong Kong, and most of the carious teeth were untreated. Caries experience was associated with dental attendance, type of primary caregiver, frequency of sugary snack intake and socio-economic background.

## Additional file

Additional file 1: Parental questionnaire: the questionnaire consisted of four parts including the child’s background information, socio-economic information, oral health–related behaviours and their parents’ dental knowledge. (DOCX 17 kb)

## Abbreviations

STROBE: the strengthening the reporting of observational studies in epidemiology
WHO: World health organization
ZINB: Zero-inflated negative binomial
