# Supplementary material for: Dental caries status and its associated factors among 5-year-old Hong Kong children: a cross-sectional study
Source: BMC Oral Health. 2017 Aug 31;17:121. doi: 10.1186/s12903-017-0413-2 (PMC5580282; doi:10.1186/s12903-017-0413-2)
Supplement: Additional file 1: — Parental questionnaire: the questionnaire consisted of four parts including the child’s background information, socio-economic information, oral health–related behaviours and their parents’ dental knowledge. (DOCX 17 kb) [file 12903_2017_413_MOESM1_ESM.docx]

| **Questionnaire (English)** |
| --- |

**Part A: Eating Habit of Your Children**

1. Is your child currently bottle-fed with milk or sugary drinks before sleeping?

□ Yes □ No

2. When does your child stop bottle-feeding with milk or sugary drinks?

□ 1 to 12 months old

□ 13 to 18 months old

□ 19 to 24 months old

□ After 24 months old

□ Others, please specify: __________________

3. Other than meals, how often does your child snack? (e.g., soft drinks, juice, snack, candy, biscuits, fruits, desserts)

□ None

□ 1 - 2 times

□ 3 - 4 times

□ 5 - 6 times

□ 7 - 8 times

□ 9 - 10 times

□ More than 10 times

**Part B: Oral Hygiene Habit of Children**

4. When did your child start brushing?

□ 1 to 12 months old

□ 13 to 18 months old

□ 19 to 24 months old

□ After 24 months old

□ Others, please specify: _____________

5. How many times does your child brush every day?

□ Never 　　□ Seldom 　　□ Once 　　□ Two times or more

6. Does anyone assist your child in brushing his/her teeth?

□ Yes　　□ No

7. Have your child ever seen a dentist?

□ Yes, with regular review □ Yes, without regular review □ Never

8. Does your child use other aids to clean his/her teeth? (e.g., mouth-rinse, dental floss)

□ Yes　　□ No

**Part C: Oral Health Knowledge**

9. The causes of dental decay include:

|  |  | Yes | No | I don’t know |
| --- | --- | --- | --- | --- |
| a) | Too much consumption of candies | □ | □ | □ |
| b) | Unclean teeth | □ | □ | □ |
| c) | Tooth worms attack | □ | □ | □ |
| d) | “Hot air” | □ | □ | □ |

10. Preventions of tooth decay include:

|  |  | Yes | No | I don’t know |
| --- | --- | --- | --- | --- |
| a) | Medicine | □ | □ | □ |
| b) | Herbal tea | □ | □ | □ |
| c) | Use of fluoridated toothpaste | □ | □ | □ |
| d) | Decrease frequency of sugar consumption | □ | □ | □ |

11. Effects of fluoride to teeth include:

|  |  | Yes | No | I don’t know |
| --- | --- | --- | --- | --- |
| a) | No effect | □ | □ | □ |
| b) | Prevent tooth decay | □ | □ | □ |
| c) | Tooth whitening | □ | □ | □ |
| d) | Prevent periodontal disease | □ | □ | □ |

12. Which of the following food can cause tooth decay?

|  |  | Yes | No | I don’t know |
| --- | --- | --- | --- | --- |
| a) | Soft drinks | □ | □ | □ |
| b) | Ice-cream | □ | □ | □ |
| c) | Cheese | □ | □ | □ |
| d) | Peanuts | □ | □ | □ |

13. The causes of gum bleeding include:

|  |  | Yes | No | I don’t know |
| --- | --- | --- | --- | --- |
| a) | Unclean teeth | □ | □ | □ |
| b) | It is a normal phenomenon | □ | □ | □ |

14. Methods to prevention of periodontal disease include:

|  |  | Yes | No | I don’t know |
| --- | --- | --- | --- | --- |
| a) | Tooth brushing | □ | □ | □ |
| b) | Saline mouth-rinsing | □ | □ | □ |
| c) | Regular scaling (professional tooth cleaning) | □ | □ | □ |

**Part D: Student Information**

15. Name of Student : _________________

16. Gender : □ M　　□ F

17. Date of birth : ____/____/______ (DD / MM / YYYY)

18. Place of birth : □ Hong Kong □ China □ Others, please specify: _________

19. Contact number : ________________ (for follow-ups)

**Part E: Other Information**

20. Home situation:

□ Both parents

□ Others

21. Monthly family income:

□ HK $ 10,000 or below

□ HK $ 10,001 - 15,000

□ HK $ 15,001 - 20,000

□ HK $ 20,001 - 25,000

□ HK $ 25,001 - 30,000

□ HK $ 30,001 - 35,000

□ HK $ 35,001 or above

22. Parent education level:

|  | Father | Mother |
| --- | --- | --- |
| Elementary school | □ | □ |
| High school | □ | □ |
| Collage or above | □ | □ |

23. Who usually take care of your child? (Please choose one)

□ Parents □ Grandparents

□ Domestic helper □ Others (e.g., relatives, friends)

~ Thank you ~
